# Development of VHH Antibodies against Dengue Virus Type 2 NS1 and Comparison with Monoclonal Antibodies for Use in Immunological Diagnosis

**DOI:** 10.1371/journal.pone.0095263

**Published:** 2014-04-21

**Authors:** Aneela Fatima, Haiying Wang, Keren Kang, Liliang Xia, Ying Wang, Wei Ye, Jufang Wang, Xiaoning Wang

**Affiliations:** 1 School of Bioscience and Bioengineering, South China University of Technology, Guangzhou, PR China; 2 Guangzhou Wondfo Biotech Co., Ltd, Scientific City, Guangzhou, PR China; 3 Shanghai-MOST Key Laboratory of Health and Disease Genomics, Chinese National Human Genome Center at Shanghai, Shanghai, PR China; 4 Institute of Life Science, General Hospital of The People’s Liberation Army, Beijing, PR China; University of Rochester, United States of America

## Abstract

The possibility of using variable domain heavy-chain antibodies (VHH antibodies) as diagnostic tools for dengue virus (DENV) type 2 NS1 protein was investigated and compared with the use of conventional monoclonal antibodies. After successful expression of DENV type 2 NS1 protein, the genes of VHH antibodies against NS1 protein were biopanned from a non-immune llama library by phage display. VHH antibodies were then expressed and purified from *Escherichia coli*. Simultaneously, monoclonal antibodies were obtained by the conventional route. Sequence analysis of the VHH antibodies revealed novel and long complementarity determining regions 3 (CDR3). Epitope mapping was performed via a phage display peptide library using purified VHH and monoclonal antibodies as targets. Interestingly, the same region of NS1, which comprises amino acids ^224^HWPKPHTLW^232^, was conserved for both kinds of antibodies displaying the consensus motif histidine-tryptophan-tryptophan or tryptophan-proline-tryptophan. The two types of antibodies were used to prepare rapid diagnostic kits based on immunochromatographic assay. The VHH antibody immobilized rapid diagnostic kit showed better sensitivity and specificity than the monoclonal antibody immobilized rapid diagnostic kit, which might be due to the long CDR3 regions of the VHH antibodies and their ability to bind to the pocket and cleft of the targeted antigen. This demonstrates that VHH antibodies are likely to be an option for developing point-of-care tests against DENV infection.

## Introduction

Dengue infection, commonly called bone breaker illness, is mosquito (*Aedes aegypti/Aedes albopictus*) borne. This disease ranges across tropical and sub-tropical regions around the world, especially in Asian and Latin American countries. The global incidence of dengue has increased 30-fold in recent decades. It was recently estimated that 50–100 million dengue infections occur each year and almost half of the world’s population lives in countries where dengue is endemic [1]–[2]. DENV belongs to the *Flaviviridae* family with four distinct serotypes (DENV1, DENV2, DENV3 and DENV4). Infection with any of these serotypes causes clinical symptoms including dengue fever, dengue hemorrhagic fever (DHF) and dengue shock syndrome (DSS). Due to the absence of a suitable vaccine, effective, early and rapid diagnosis against all serotypes of DENV is of significance to reduce the morbidity and mortality of DHF and DSS, especially in developing countries [3]–[4]. DENV contains a single open reading frame of approximately 11 kb with three structural (C, prM and E) and seven non-structural proteins (NS1, NS2A, NS2B, NS3, NS4A, NS4B and NS5) [5]. NS1 is one of the most important nonstructural glycoproteins (40–50 kDa) and is considered to be immunogenic either secreted as a soluble hexamer form or as a membrane-associated protein on DENV infected cells [6]–[7]. NS1 represents an interesting target antigen for diagnosis due to its presence in the blood of infected patients mostly from days 1–6 after the onset of clinical symptoms and in significant amounts from days 6–10 in both primary and secondary infection [8]–[9].

The diagnosis of dengue infection has always been a great challenge due to the short life cycle of the virus. Many methods have been explored and used to diagnose dengue infection, including virus isolation [10], viral RNA direct detection [4], virus-specific IgM antibody detection [11], antigen capture enzyme linked immunosorbent assays [12]–[13] and immunochromatographic assays [14]. Among these methods, the gold nanoparticle-based immunochromatographic assay has drawn much attention as a promising tool for the development of a biosensor for early and rapid detection of the disease. Usually, monoclonal antibodies (MAbs) are used for immunochromatographic assays [14]–[16]. Although there are many advantages of MAbs in biomedical research, there are also several limitations, such as the long time period and effort required, especially in the cloning and selection process, to obtain a successful monoclonal antibody.

The discovery of variable domain heavy-chain antibodies (VHH antibodies or nanobodies) of the *Camelidae* family by Hamers–Casterman launched a new era providing valuable ligands for diagnosis, imaging and therapy that are better than monoclonal antibodies [17]. Nanobodies are the smallest intact antigen-binding fragments (around 15 kDa) that have full antigen-binding capacity in the absence of light chains due to their long CDR3 and good shelf-life [18]–[20]. These new VHH domains with their versatile structural and functional properties form the basis of a new generation of antibodies for the diagnosis of infectious diseases as they bind to the pocket and cleft of the targeted antigen [21]. So it is highly likely that nanobodies will be potential tools in the development of biosensors based on immunochromatographic assays.

The generation of serotype-specific antibodies makes it possible to identify serotype-specific epitopes that can be used to investigate the mechanism of NS1-mediated immunologic functions, diagnosis and vaccine development [22]. A phage library displaying different peptide sequences exposed to targets (antibodies) and the elution of specifically bound phages can provide the information of fragments binding with the targets [23].

The objectives of the present study are to screen and purify nanobodies against recombinant NS1 protein of DENV type 2 from a non-immune llama (*Lama glama*) library; determine the binding epitopes of VHH antibodies using the phage displaying peptides library (Ph.D12 kit); and investigate the possibility of developing VHH antibodies as a diagnostic tool as an alternative to conventional monoclonal antibodies.

## Materials and Methods

### Ethics Statements

The study was approved by the Institutional Review Board (IRB) of the National Engineering Laboratory of Point-of-Care Tests, Wondfo, Guangzhou, China (permit number: 20110503). The specific approval number for experimental animal use was SCXK (Yue) 2011-002. Blood samples of patients used for the experiments were collected from No. 8 Provincial People’s Hospital, Guangzhou, China. All participants provided written informed consent for the use of the clinical samples in this study and this consent procedure was also approved by the Ethics Committee of No. 8 Provincial People’s Hospital, Guangzhou, China (permit number: 20100431).

### Preparation of Recombinant NS1 Protein (rNS1)

The genomic RNA was extracted from a DENV type 2 strain NGC-infected C6/36 mosquito (*Aedes albopictus*) cell culture (kindly provided by Zhujiang Hospital, Guangzhou, China) and used as a template for RT-PCR with forward primer (5′-TTA**CATATG**GATAGTGGTTGCGTTGTGAGC-3′) and reverse primer (5′-TTA**GTCGAC**GGCTGTGACCAAGGAGTTGA-3′). The amplified NS1 gene, which showed 98% nucleotide sequence identity to that of DENV type-2 NGC strain NS1 (NCBI GenBank M29095.1), was ligated into the *NdeI* and *SalI* sites of the expression vector pET-30a (+) (Novagen,Madison, WI) in the open reading frame and downstream of the His-tag coding sequence. The plasmid pET-30a (+)-NS1 was transformed into *E. coli* BL21 (DE3). The rNS1 protein was expressed as inclusion bodies and then purified using a previously described method [24]. The identity of the rNS1 protein was confirmed by SDS-PAGE, western blot analysis and indirect ELISA with rabbit polyclonal antibody against NS1.

To investigate whether the rNS1 protein re-folded correctly, circular dichroism (CD) was detected using a Chirascan Circular Dichroism Spectropolarimeter (UK) with a quartz cuvette (0.01-cm path length), and spectra from scans at 30 nm/min speed were averaged. A Varian Cary Eclipse spectrofluorometer (Australia) was used for fluorescence spectroscopy measurements at 25°C. The excitation wavelength was 278 nm and the emission spectrum was recorded from 300–420 nm [25].

### Preparation of Monoclonal Antibodies (MAb)

Four to 5-week-old female BALB/c mice were immunized subcutaneously with 100 µg rNS1 emulsified with complete Freund’s adjuvant (Sigma-Aldrich), followed by three booster doses of 50 µg antigen (rNS1) emulsified with incomplete Freund’s adjuvant at 10-day intervals. Three days before cell fusion, a booster dose of 100 µg rNS1 in saline was injected intravenously every day. Hybridomas were produced by fusing spleen cells from the immunized mice (sacrificed by CO_2_ inhalation, a recommended method of euthanasia) with myeloma SP2/0-Ag14 cells [26]. Positive hybridomas were selected by indirect ELISA with rNS1 as coating antigen and cloned by limiting dilution. Monoclonal antibodies were purified from ascites using a HiTrap Protein G HP affinity column (GE Healthcare) according to the manufacturer’s instructions. The identification and activity of anti-rNS1 monoclonal antibodies were confirmed by SDS-PAGE, western blot analysis and indirect ELISA.

### Indirect ELISA

The binding affinity of anti-rNS1 monoclonal antibodies was confirmed by indirect ELISA. A 96-well microtiter plate was coated overnight at 4°C with 2 µg/ml (100 µl/well) rNS1 antigen and malarial LDH recombinant protein (expressed in our laboratory) to check the cross reaction. The plate was washed once with PBST (0.5% Tween20) followed by blocking with 3% BSA in PBST (200 µl/well) at 37°C for 2 h. Serial dilutions (10^−3^ to 10^−6^-fold) of 3B3 monoclonal antibody (2 mg/ml) were prepared in antibody dilution buffer (1% BSA in 1×PBS with 0.5% Tween20) and 100 µl/well were added after washing the plate once. A positive control of anti-DENV2-rNS1 mouse monoclonal antibody was provided by Wondfo (Guangzhou, China). The plate was then incubated at 37°C for 1 h and washed three times with PBST. HRP-conjugated goat anti-mouse IgG (ZSGB-BIO, Beijing, China) diluted 1∶10,000 was added (100 µl/well) for detection and incubated at 37°C for 30 min. 3,3′,5,5′-Tetramethylbenzidine solution with hydrogen peroxide (100 µl/well) was used as the substrate after washing the plate three times and incubated at 37°C for 10 min in the dark. The reaction was stopped by 2 M H_2_SO_4_ (50 µl/well) and the plate was immediately read using an ELISA reader at OD_450_.

### VHH Antibody Screening and Phage Binding Analysis

Biopanning was performed as described previously with slight modifications [27]. Briefly, four rounds of biopanning were performed. Non-immune llama single domain heavy chain (VHH) phage displaying the antibody library (kindly provided by Chinese National Human Genome Center at Shanghai, Shanghai, China) was subjected to 2×yeast extract and tryptone growth medium (2×YT) supplemented with ampicillin (100 µg/ml) and glucose (2%w/v). The library stock was incubated at 37°C, 220 rpm until the OD_600_ reached 0.5. It was then infected with M13K07 helper phage (GE Healthcare) and amplified overnight with the addition of kanamycin (50 µg/ml) and IPTG (0.1 mmol/l) at 30°C, 220 rpm. The phage particles from the culture supernatant were precipitated with PEG (20% polyethylene glycol 6000, 0.5 M NaCl, resuspended in sterile PBS) on ice for 1 h. The pellet was resuspended in 5 ml TE buffer after centrifugation and used for the next panning.

Nunc-Immuno Maxisorp tubes (Fisher Scientific) were coated overnight at 4°C with different concentrations (10, 5, 2.5 and 1 µg/ml) of target rNS1 antigen for rounds 1, 2, 3 and 4, respectively. The reaction was blocked with 2% BSA for 1 h at 37°C. The phages from each round of panning were also pre-incubated with the same blocking solution for 30 min at 37°C and added to the immunotubes coated with antigen. The immunotubes were then incubated at 37°C for 1 h. Unbound phages were removed by intensive washing in each round. The bound phage was eluted with 0.1 M HCl-glycine (pH 2.2) after gently shaking at room temperature for 10 min and then neutralized with 1 M Tris-HCl (pH 7.4). At the end of each round of panning, the eluted phages were used to infect *E. coli* TG1 cells for amplification. After centrifugation at 5000 rpm for 5 min at 4°C, the pellet was resuspended with 1 ml of 2×YTAG medium and then spread on the plates containing 2×YTAG agar medium. The plates were incubated overnight at 37°C. Colonies were scraped from the plates and used as the library for the next panning.

After the fourth round of biopanning, 96 colonies were randomly picked and cultured in 2×YTAG medium in a U-bottom 96-well plate (Nunc, Denmark) to determine the reactivity of VHH-phages towards rNS1. When the OD_600_ reached 0.5, the colonies were infected with 20-fold M13K07 and amplified overnight with the addition of kanamycin (50 µg/ml) and IPTG (0.1 mmol/l) at 30°C, 220 rpm. Culture supernatant was added to the ELISA microplate (100 µl/well) coated with rNS1 antigen (1 µg/ml) and blocked with 3% BSA. After washing, bound phages were detected with HRP-conjugated anti-M13 mouse monoclonal antibody (GE Healthcare). Positive clones were selected by OD_492_-tested clone/OD_492_-negative control >2.1 (generally considered as the threshold value), meanwhile the negative control was a blank. The positive clones were then sequenced by Shanghai HuaGene Biotech Co. Ltd. (Shanghai, China). Sequence analysis of plasmids (pCANTAB5E-VHH) and alignment were performed by DNAMAN, primer-primer (version 5) software, CLUSTALW and IMGT (international ImMunoGeneTics) information system.

### VHH Antibody Expression and Purification

After double digestion with *BamHI* and *HindIII* enzymes, the VHH genes from the selected clones were ligated into pET-22b (+) vector and transformed into *E. coli* BL21 (DE3) (Novagen, Madison, WI). Large scale production of recombinant VHHs was performed by culturing the bacteria in LB medium supplemented with ampicillin (1∶1000) until the OD_600_ reached 0.6–0.8. Then, to induce VHH expression, IPTG was added to a final concentration of 1 mM and the culture was incubated at 37°C, 220 rpm for 4–5 h. Cells were harvested and the pellet was resuspended in lysis buffer (50 mM NaH_2_PO_4_, 300 mM NaCl, 10 mM imidazol, pH 8, 1 ml of 30 µg/ml lysozyme) followed by ultrasonication. The lysate was centrifuged at 10,000 rpm, 4°C for 10 min. The supernatant was loaded onto a Ni-nitrilotriacetic acid (Ni-NTA) superflow sepharose column (Qiagen, Hilden, Germany) and the VHH antibody was eluted with elution buffer (50 mM NaH_2_PO_4_, 300 mM NaCl, 250 mM imidazol).

### Surface Plasmon Resonance

The binding kinetics and affinity of the VHH antibodies and monoclonal antibody for the rNS1 antigen were analyzed by real-time surface plasmon resonance (SPR) using Plexera V1. Both kinds of antibodies in 10 mM sodium acetate buffer (pH 4.5) were covalently immobilized on a three dimensional sensor chip (Plexera, Guangzhou, China) using EDC/NHS activation. All analyses were performed with a flow rate of 2 µl/s at 22°C. At the end of rNS1 injection, the running buffer (10 mM HEPES, 0.3 M NaCl, 3.4 mM EDTA, 0.05% Tween) flowed for 500 s, followed by the regeneration of the three dimensional chip using 1000 µl of 10 mM NaOH. Binding kinetics was evaluated using a 1∶1 Langmuir binding model.

### Preparation of Gold Nanoparticle-conjugated Antibodies and the Immunochromatographic Test Strips

Monoclonal antibody and VHH antibody immobilized rapid diagnostic kits were developed in this study. Colloidal gold was prepared by following a previously reported method with slight modifications [16]. Colloidal gold-conjugated 3 M4 mouse monoclonal antibody against DENV type 2 rNS1 was provided by Wondfo (Guangzhou, China) and used as the detection antibody in both types of kits. This 3 M4 monoclonal antibody was immobilized on polyester fiber for the preparation of a conjugated pad. Monoclonal antibody and VHH antibody against DENV type 2 rNS1 developed in this study were immobilized on the nitrocellulose (NC) membrane as capture antibodies to give the test line (T line), which was the most important part of the strip. Goat anti-mouse IgG antibody specific to the detection antibody was also immobilized on the NC membrane to produce a control line (C line). All of these components along with a sample pad and absorbent pad were pasted onto a PVC board. The whole assembled board was cut into 3-mm wide strips. The sample was dropped onto the sample pad and allowed to migrate.

After the antigen-antibody reactions, a red color caused by the accumulation of the colloidal gold at the test and control lines appears on the membrane within 10–15 min for positive samples [16]. The color intensity of each test line was compared with a standard color test card developed by Wondfo (Guangzhou, China). The analytical limit of detection was determined as the lowest concentration of rNS1 required to produce positive results by the appearance of a T line. The sensitivity and specificity of the two types of strips were determined by using dengue positive viral culture supernatants and clinical serum samples of different endemic diseases, respectively.

### Phage-display Biopanning for Epitope Mapping

The phage peptide library kit (Ph.D 12 kit, New England Biolabs, USA), which displays 12-residue peptides with random sequences fused with the N terminus of the minor coat protein (pIII) of M13 phage, was used for screening the MAb and VHH antibodies according to the manufacturer’s instruction. For each selection cycle, 2×10^11^ phages were applied to a 96-well plate pre-coated with monoclonal or VHH antibodies (100 µg/well). The level of precise phage enrichment was calculated as the input-output ratio. Three rounds of biopanning were performed. The concentration of coating antibodies (VHH and monoclonal antibodies) gradually decreased in the second and third panning cycles (50 µg/ml and 25 µg/ml, respectively) and the concentration of Tween20 in TBS increased from 0.1% to 0.5% in the second and third rounds of panning during the washing steps. After the last panning, unamplified phage eluate was titrated onto LB medium plates containing IPTG and X-Gal. Plaques were randomly picked for each antibody and amplified. Then, single stranded phage DNA from each clone was isolated for sequencing with the −96 gIII sequencing primer 5′-CCCTCATAGTTAGCGTAACG-3′ and used to deduce the amino acid sequences of encoded 12-mer peptides. The peptide sequences were aligned with the amino acid sequence of rNS1 using DNAMAN and DNASTAR software.

### Statistical Analysis

ELISA absorbance data were analyzed using analysis of variance (ANOVA) and means were compared by the Student–Newman–Keuls test [28]. Clinical data were expressed with 95% confidence intervals (CIs) as suggested by Tjitra et al. [29].

## Results

### Expression, Purification and Refolding of rNS1

**Figure 1:**
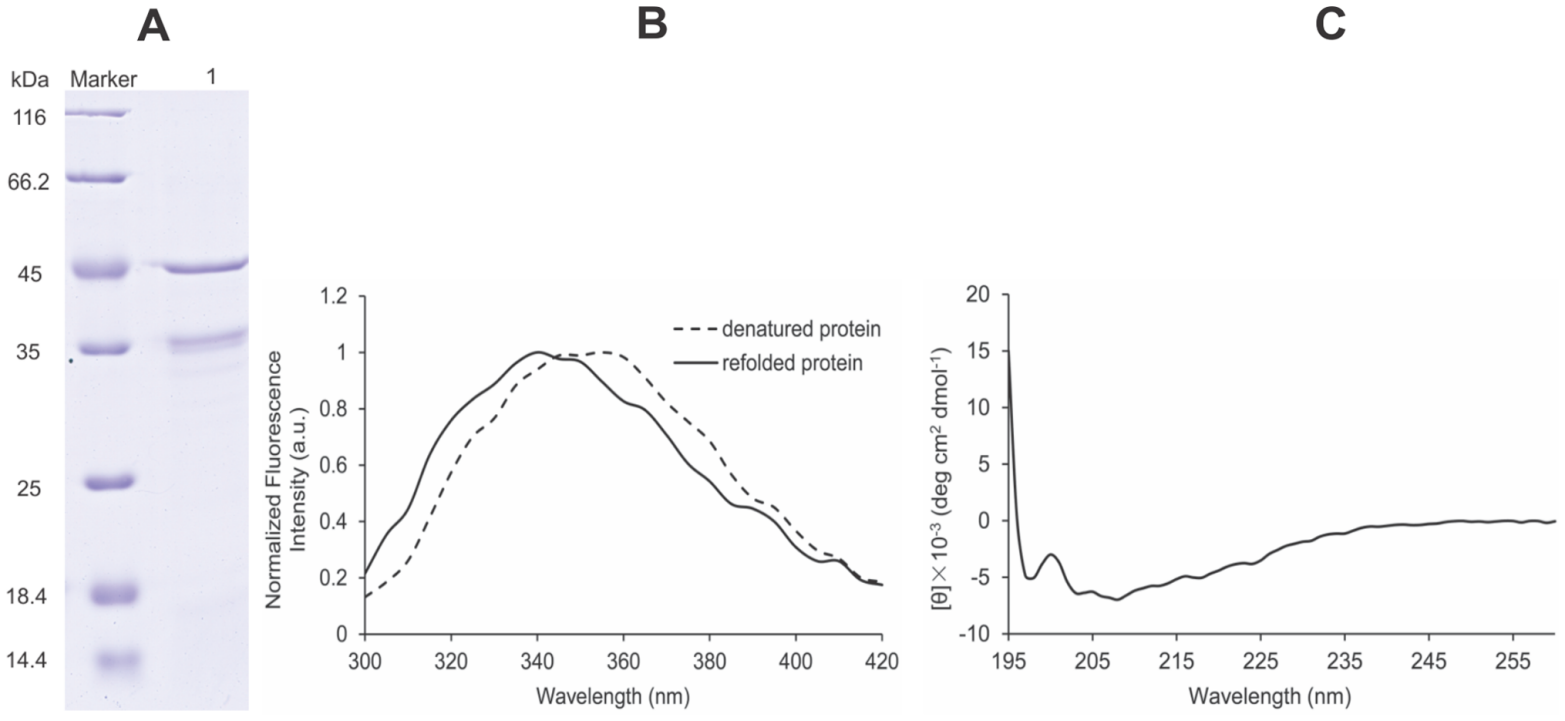
Analysis of DENV2-rNS1 refolded protein. (A) SDS-PAGE of refolded rNS1 protein, M: protein marker, lane 1: protein after refolding. (B) Correct refolding of rNS1 by fluorescence spectroscopy. The dashed line represents denatured protein with a peak at 355 nm whereas the solid line represents refolded protein with a peak at approximately 340 nm. The emission spectrum was recorded from 300 nm to 420 nm by fixing the excitation wavelength at 278 nm. (C) The CD of refolded rNS1. The experiment was performed at 20°C and samples were scanned at 30 nm/min.

The DENV2 NS1 gene was cloned into the expression vector pET-30a (+) in the correct open reading frame. The transformed BL21 (DE3) bacterial cells expressed a high level of rNS1 protein as inclusion bodies. The inclusion bodies were dissolved in denaturation buffer and purified rNS1 protein was obtained using a HiTrap Ni^2+^ column. The recovery rate of recombinant protein was up to 82%. A protein band with molecular mass of approximately 45 kDa was observed by SDS-PAGE (Figure 1A) and Western blot (data not shown). After refolding, rNS1 was analyzed by fluorescence spectroscopy of the tryptophan residue to verify proper rNS1 refolding [34]. One peak at 340 nm in the emission spectrum indicated correct refolding whereas a peak at 355 nm was observed in the emission spectrum of denatured rNS1 (Figure 1B). The CD spectrum indicated one positive peak at 192 nm and two negative peaks near 208 nm and 222 nm, indicating a mixture of secondary structure components (Figure 1C). According to the CDSSTR decomposition algorithm, rNS1 is composed of 15% α-helices, 48% β-sheets and 37% random coils/turns. The same results were also obtained by Allonso et al. [34].

### Generation of MAb and VHH Antibodies Against rNS1

**Figure 2:**
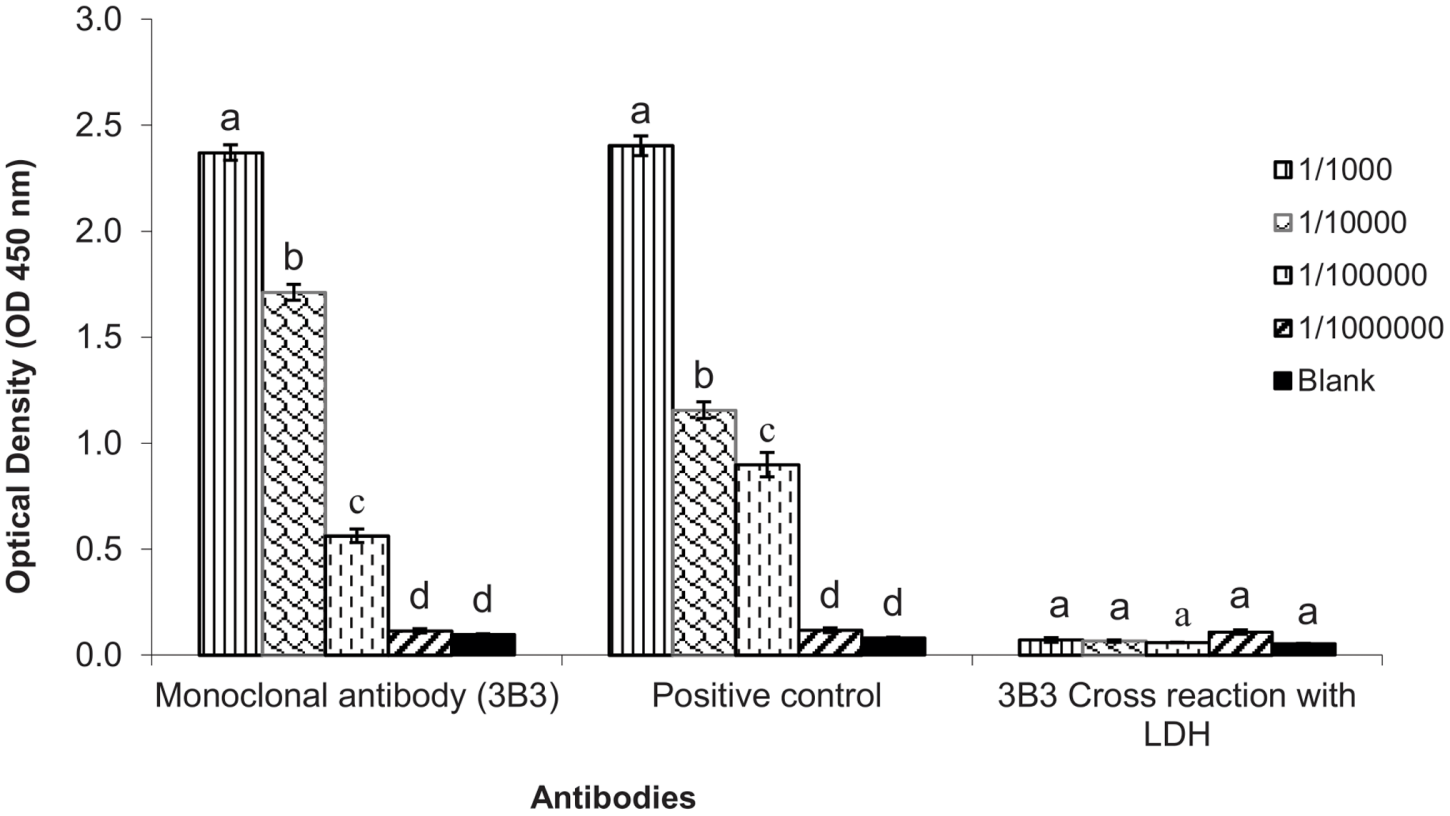
Antibody titer of monoclonal antibody (3B3) against rNS1 by indirect ELISA. The positive control was another mouse monoclonal antibody against dengue type 2 rNS1 provided by Wondfo (Guangzhou, China). Values are the means of three replicates. Mean ± SE values with the same letter within a column are not significantly different (Student–Newman-Keuls test; *P<*0.05).

Serotype-specific monoclonal antibodies and VHH antibodies against DENV type 2 rNS1 were generated. For monoclonal antibody production, in total 18 mouse hybridoma cell lines stably producing MAbs were initially established in this study on the basis of their positive reactivity with rNS1 protein after indirect ELISA. 3B3 MAb was selected for comparison with VHH antibody because it has the highest binding affinity (OD_450_ 2.372±0.037) with DENV2-rNS1 and no cross reaction was observed with His-tagged malarial LDH recombinant protein after indirect ELISA (Figure 2).

**Figure 3:**
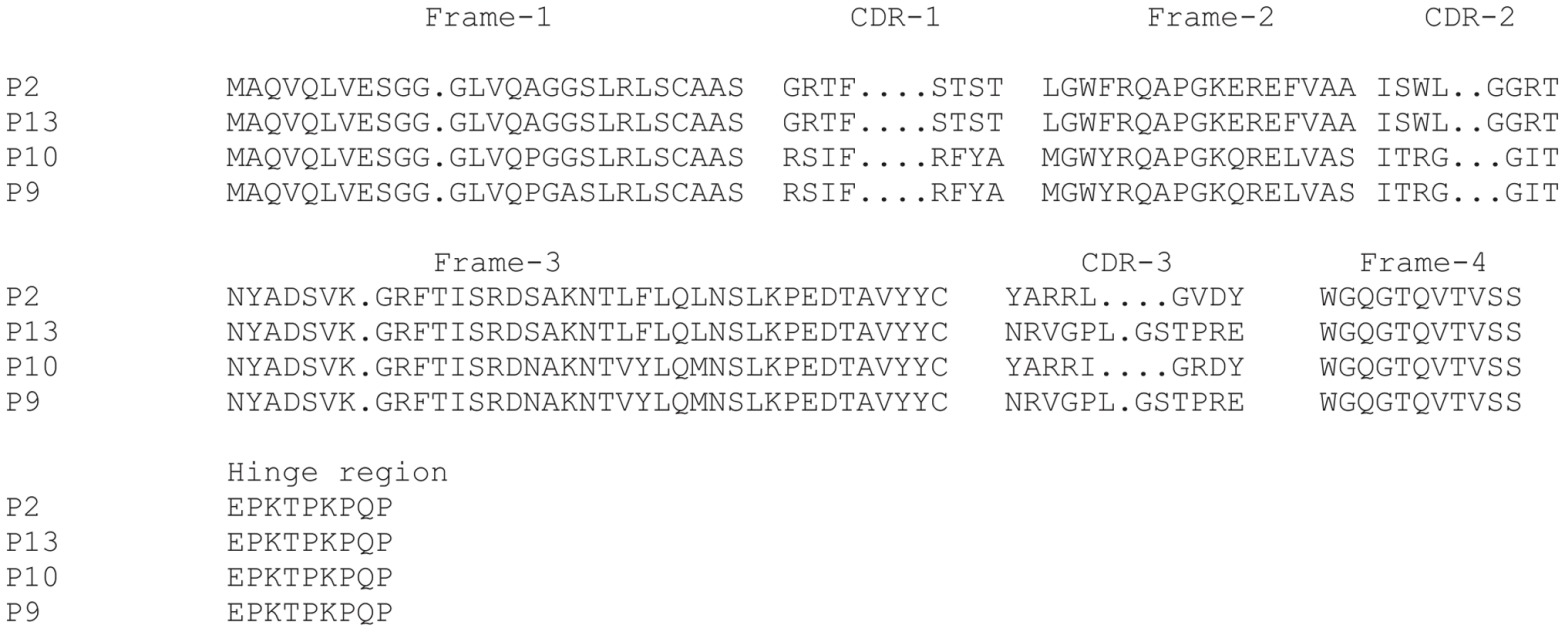
Amino acid sequence analysis of four different strains of VHH antibodies (P2, P13, P10 and P9). Complementarity determining regions (CDR), framework regions (FR) and dot gaps are defined and numbered according to the IMGT unique numbering system (GenBank accession number: KF193086–KF193089).

Fast, reliable, controllable selection and screening of single domain antibodies (VHHs) against DENV2-rNS1 protein from the non-immune llama VHH library were derived in the present study. After four rounds of biopanning and indirect ELISA, 20 positive clones were selected for sequencing. Sequence alignment and analysis revealed that most of the clones have sequence homology (data not shown). Hence, four different strains of VHHs were obtained and those designated as P2, P9, P10 and P13 were selected for further analysis. The four VHHs were analyzed for CDR and framework regions according to the IMGT unique numbering system [30] (http://www.imgt.org/3Dstructure-DB/cgi/DomainGapAlign.cgi). These VHHs have 82–87% nucleotide identity with *Lama glama* (IGHV1S3*01 and IGHJ4*01). P2 and P10 clones have nine unique nucleotides whereas P9 and P13 clones have 12 unique nucleotides in the CDR3 regions (Figure 3) (GenBank accession number: KF193086-KF193089). The long CDR3 regions indicate that those VHHs may have good binding affinity with antigen [31]–[32].

**Figure 4:**
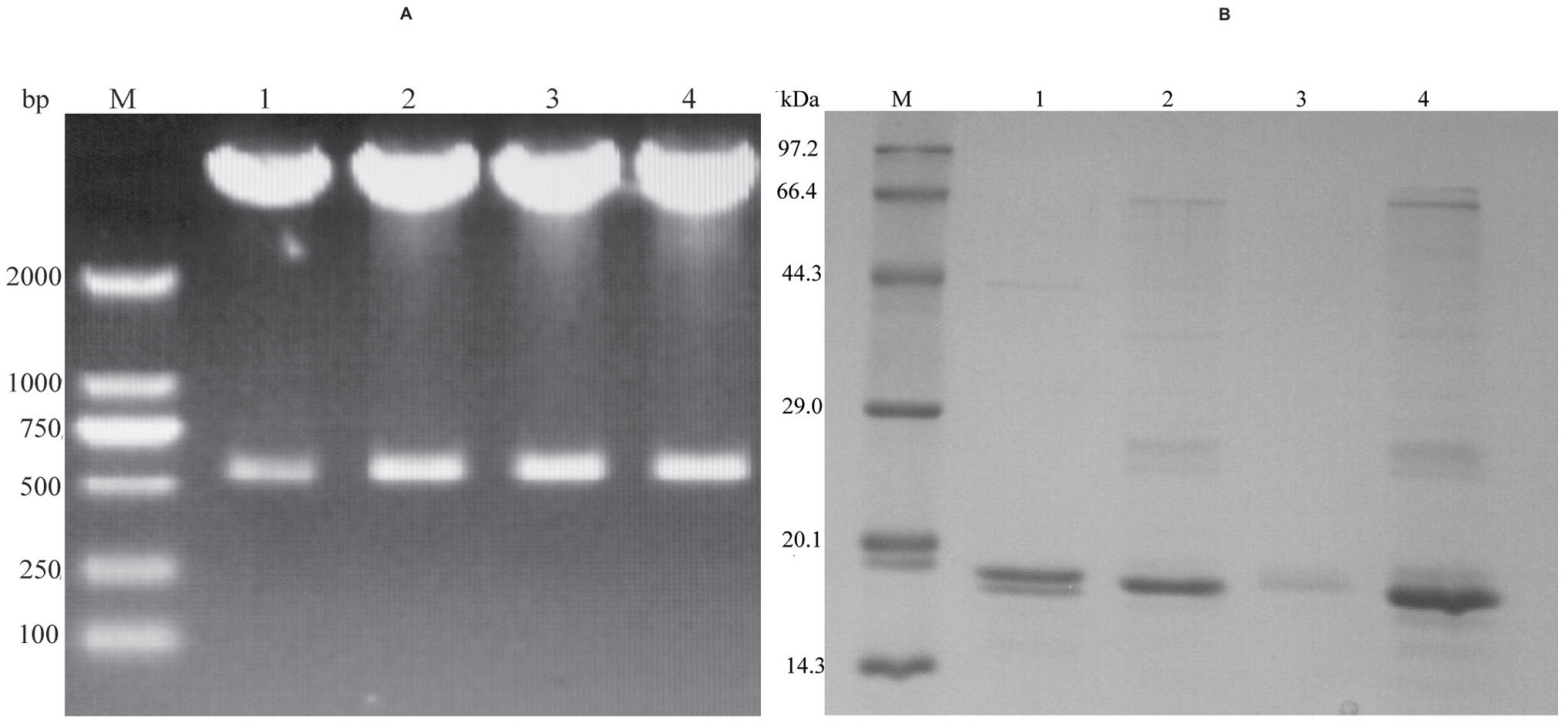
Double digestion and purification of VHH antibodies. (A) Agarose gel electrophoresis of VHH antibodies double digested with *BamHI* and *HindIII*. Upper bands of 4500 bp correspond to vector pCANTAB5E whereas lower bands of 400 bp correspond to VHH fragments of P2 (lane 1), P9 (lane 2), P10 (lane 3), P13 (lane 4), respectively, and M stands for marker. (B) 12% polyacrylamide SDS electrophoresis gel of VHH antibodies purified from *E. coli* BL21 using a Ni-NTA column. A single band of about 15 kDa is observed for each purified VHH: P2 (lane 1), P10 (lane 2), P9 (lane 3), P13 (lane 4); M stands for marker.

VHH genes (400 bp) isolated after double digestion (Figure 4A) were cloned into pET-22b (+) vectors (Novagen) and transformed into *E. coli* BL21 (DE3). Recombinant proteins were produced at high levels within a range of 5–8 mg/l from bacterial culture. P2 and P13 VHHs were expressed as inclusion bodies while P9 and P10 VHHs were expressed in soluble form. The purified proteins were analyzed by SDS-PAGE and clear bands at 15–16 kDa were obtained (Figure 4B). As the expression of P9 VHH antibody was very low, P2, P10 and P13 VHH antibodies were selected for the binding affinity test.

### Binding Affinity Analysis of VHH and Monoclonal Antibodies with rNS1

**Figure 5:**
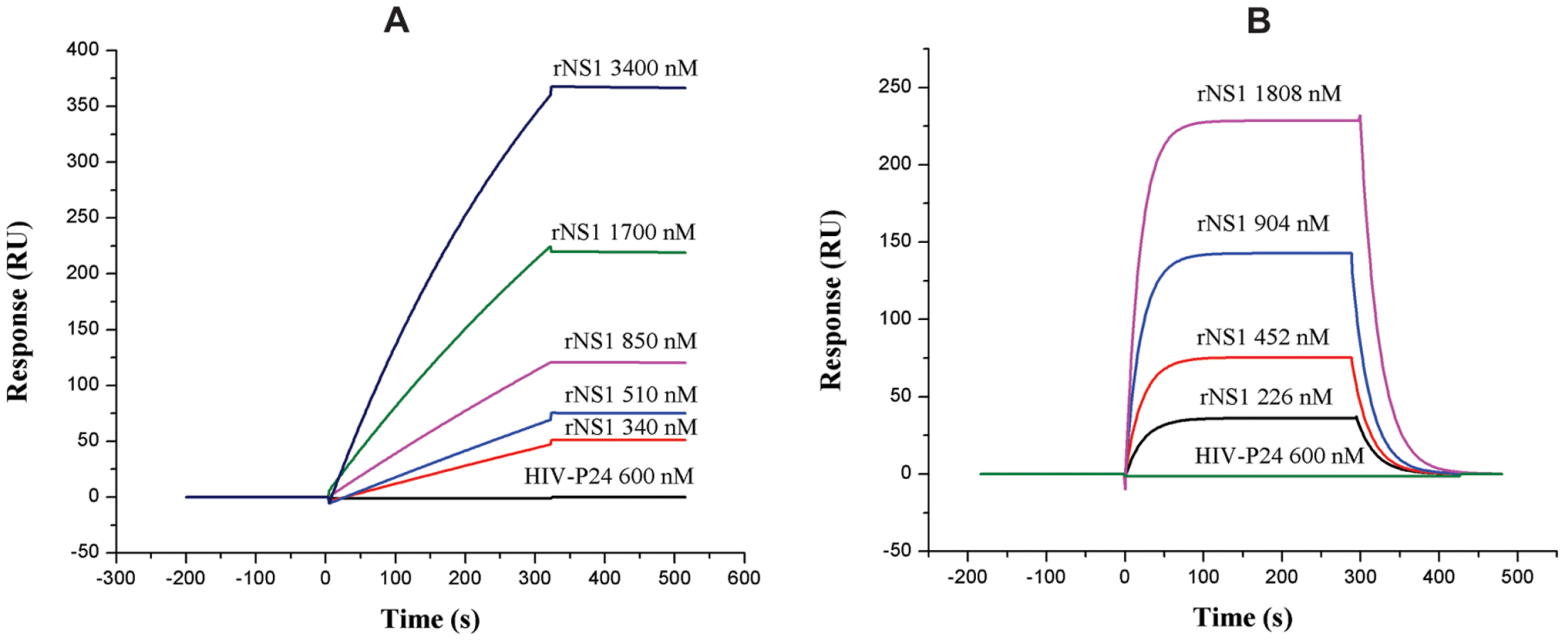
SPR sensorgrams of VHH antibody P2 and monoclonal antibody 3B3. (A) Sensorgram showing association and dissociation of ligand P2 to analytes rNS1 and recombinant HIV-p24. (B) Sensorgram showing association and dissociation of ligand 3B3 to analytes rNS1 and recombinant HIV-p24.

The results of real-time SPR using Plexera V1 revealed that these two kinds of antibodies showed good affinity for rNS1. The data output was characterized as the value of the observed response units (RU) from the sample cells minus the RU from the reference cell (Figure 5). The dissociation constant (K_D_) was evaluated from the kinetics parameters of association (k_a_) and dissociation rate constants (k_d_) by fitting the data using the BIA evaluation 3.1 software (GE Healthcare). The K_D_ value of the P2 VHH antibody was 2.79×10^–8 ^M, which was within the range of K_D_ values of most VHHs for their target antigens [33]–[35], while P10 and P13 had lower binding affinities (data not shown). The K_D_ value for the 3B3 monoclonal antibody was 5.74×10^–6 ^M (Table 1), which was nearly the same as some other studies [14], [36]. Furthermore, both of antibodies did not display cross reaction with other His-tagged HIV-p24 recombinant proteins (expressed in our laboratory). Based on these findings, P2 VHH antibody was assumed to be the most suitable for comparison with 3B3 MAb in further studies (rapid diagnostic tests and epitope mapping).

**Table 1:** Kinetic constants for monoclonal antibody (3B3) and VHH antibody (P2) against the recombinant protein NS1.

Antibody	k_a_ (M^−1^ s^−1^)	k_d_ (s^−1^)	K_D_ (k_d_/k_a,_ M)
3B3	4.62×10^3^	2.65×10^−2^	5.74×10^−6^
P2	7.34×10^2^	2.05×10^−5^	2.79×10^−8^

### Development of Rapid Diagnostic Kits for Dengue Disease

The pairing of colloidal gold-conjugated 3 M4 monoclonal antibody as detection antibody with 3B3 MAb as well as P2 VHH antibody as capture antibodies gave the best combination for the preparation of kits. P10 and P13 VHH antibodies could not be paired with 3 M4 detection antibody. Dengue type 2 viral culture supernatants, non-dengue serum specimens of different unknown diseases and serum from a healthy human were used for the evaluation of VHH antibodies and monoclonal antibodies immobilized kits.

**Figure 6:**
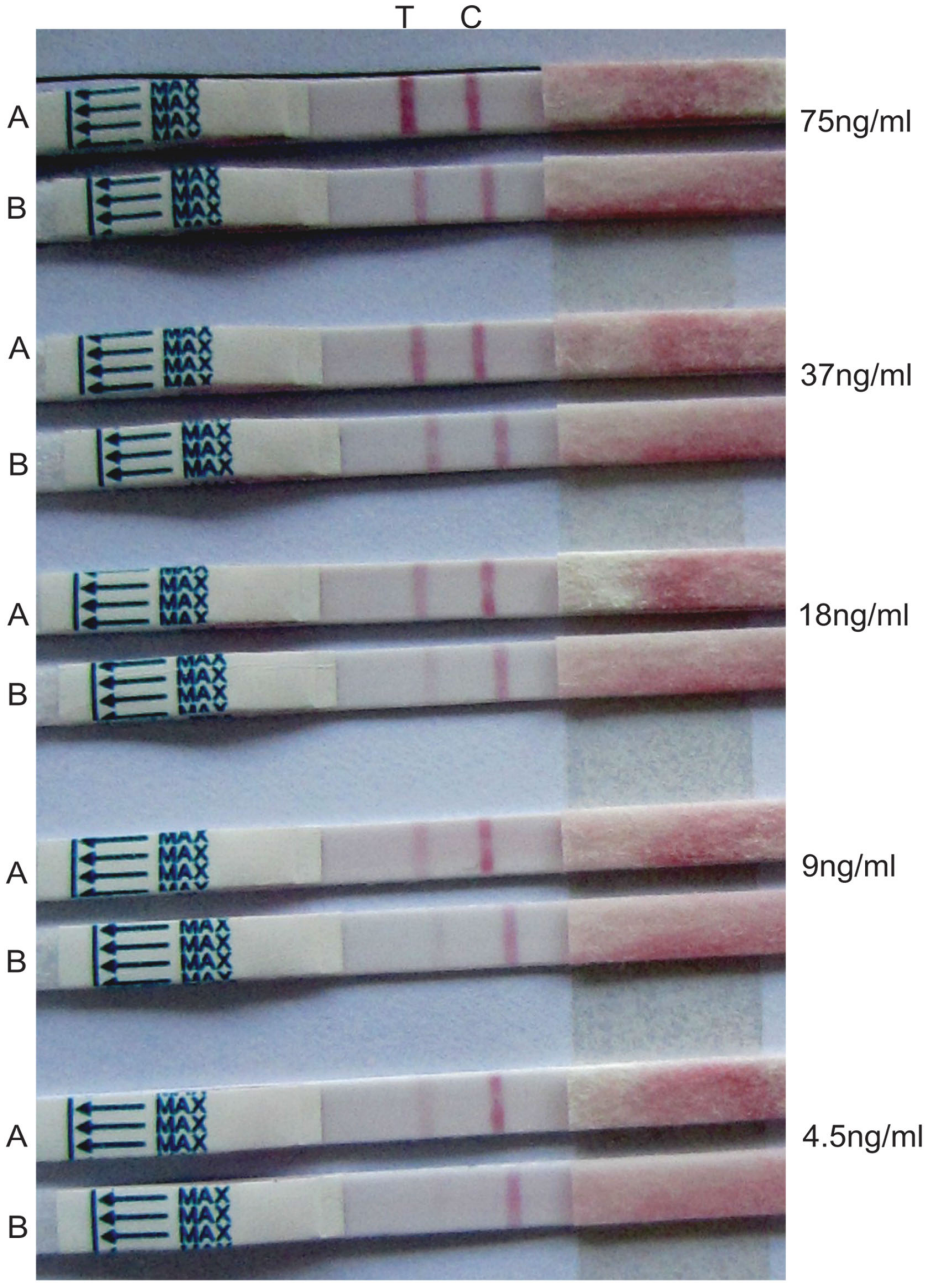
Sensitivity of antibody immobilized kits to rNS1 antigen. Test strips were treated with different concentrations of rNS1 antigen. Upper strip (A) was VHH antibody immobilized while lower strip (B) was monoclonal antibody immobilized at each tested concentration. T and C represent test and control lines, respectively. Detection limits visually observed were 4.5 ng/ml and 9 ng/ml for VHH and monoclonal antibodies immobilized strips, respectively.

For analytical sensitivity, different dilutions of rNS1 (75 ng/ml, 37 ng/ml, 18 ng/ml, 9 ng/ml and 4.5 ng/ml) in 0.85% NaCl were tested on both kinds of strips to determine the limit of detection, which is the lowest concentration of recombinant NS1 antigen required to produce positive results. For this recombinant NS1 protein of dengue type 2, 4.5 ng/ml and 9 ng/ml detection limits were observed visually for the VHH antibody (P2) immobilized kit and monoclonal antibody (3B3) immobilized kit, respectively. The test lines for every concentration of antigen in the VHH antibody immobilized strips were a little darker than those in the monoclonal antibody immobilized strips (Figure 6). To evaluate the sensitivity, inactivated viral culture supernatant of dengue type 2 containing native NS1 was used because dengue type 2 positive blood samples were not available from any hospital in Guangzhou, China. This viral culture supernatant was diluted in ratios of 1∶10, 1∶20, 1∶100, 1∶150 and 1∶200 in 0.85% NaCl and tested on both types of strips. A parallel experiment was performed with commercially available SD Bioline Dengue Duo (NS1/IgM/IgG) kits (Kyonggi-do, Korea) to compare and check the accuracy of diagnosis of the kits prepared in the present study. The color intensity of the T line was compared with a standard color test card developed by Wondfo (Guangzhou, China). Strong detection levels based on the color of the T line were observed for dilutions 1∶10 and 1∶20, but the level gradually became weaker for other dilutions (Table 2). The VHH immobilized strips displayed higher sensitivity with the appearance of darker colored T lines for each dilution as compared to the monoclonal antibody immobilized strips and the commercial kit. The native NS1 detection level of the monoclonal antibody immobilized kit prepared in the present study was consistent with that of the commercial kit. The end point dilution/limit dilution of detectable NS1 in the viral culture supernatant was 1∶150 for the monoclonal antibody immobilized kit and the commercial kit. But for the VHH antibody immobilized kit, the end point dilution was 1∶200. The color of the T line was weak and difficult to identify at the limit of dilution. The sensitivity indicated that the performances of both kits prepared in the present study were accurate. Thus, VHH antibody immobilized kit exhibited a higher sensitivity with a 1∶200 dilution limit as compared with our monoclonal antibody immobilized kit and the commercial kit with dilution limits of 1∶150.

**Table 2:** Sensitivity and specificity of monoclonal and VHH antibodies based on immunochromatographic tests.

	Test strip based on VHH	Test strip based on MAb
Sensitivity (viral culturesupernatant dilutions)	Color of T line	Color of T line
1∶10	++++	+++
1∶20	+++	++
1∶100	++	+
1∶150	+	±
1∶200	±	−
Specificity (95% CI)(Non-dengue samples)	99.50% (97.25 to 99.92)	95.24% (91.41 to 97.69)

“++++” stands for high detection level with strong color band; “+++” less strong;

“++” medium; “+” light color band; “±” limit dilution; “−” absent.

The specificities of the P2 VHH antibody and the 3B3 MAb immobilized kits were measured using a panel of 100 non-dengue samples at two different times (fresh samples of different diseases were taken from hospital each time). Parallel experiments for specificity were also performed with SD Bioline Dengue Duo (NS1/IgM/IgG) commercial kits (Kyonggi-do, Korea) for comparison. The specificity (95% CI) of the VHH immobilized rapid diagnostic strip was 99.50% (95% CI: 97.25% to 99.92%) with one false positive result. The monoclonal antibody immobilized strip gave ten false positive results, so its specificity (95% CI) was 95.24% (95% CI: 91.41% to 97.69%). The specificity (95% CI) of the Korean commercial kit was 98.04% (95% CI: 95.05% to 99.45%), which was higher than the monoclonal antibody immobilized kit but lower than the VHH immobilized kit. Hence, the VHH antibody immobilized kit showed a higher specificity than our monoclonal antibody immobilized kit and the Korean commercial kit. These results indicate that the overall performance of the VHH antibody immobilized rapid diagnostic kit was better than that of the monoclonal antibody immobilized kit.

### Epitope Mapping

**Figure 7:**
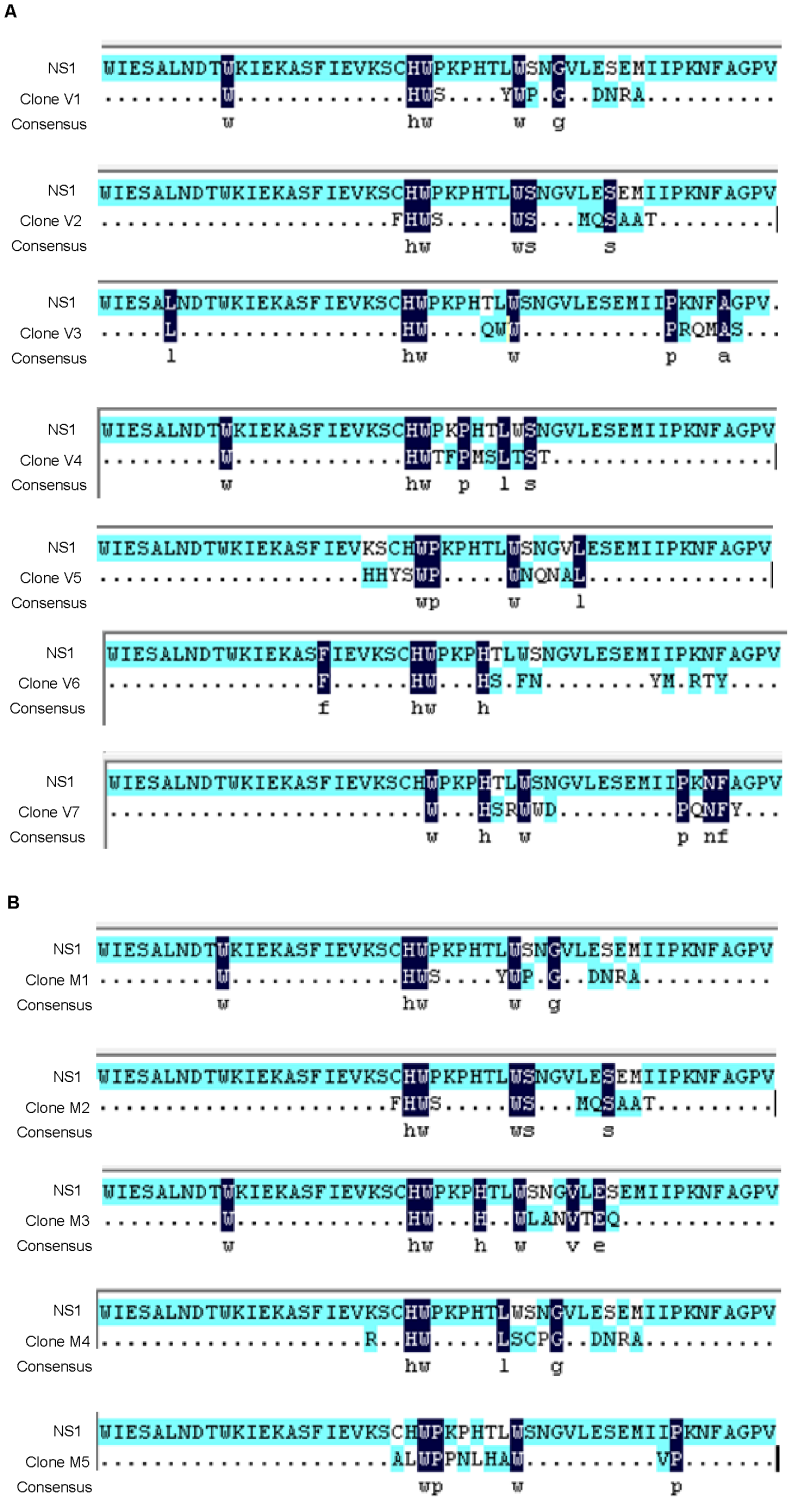
Sequence similarity between binding peptides and dengue type2-rNS1. (A) Clones V1–V7 represent different binding peptides of VHH antibody (P2). All selected binding peptides were homologous to the ^224^HWPKPHTLW^232^ amino acid region of rNS1. (B) Clones M1–M5 represent different binding peptides of monoclonal antibody (3B3). All selected binding peptides were homologous to the ^224^HWPKPHTLW^232^ amino acid region of rNS1.

As mentioned above, only P2 VHH antibody was suitable for the development of rapid diagnostic kits. Therefore, P2 antibody was selected for epitope mapping and compared with 3B3 MAb to determine whether different or similar epitopes were involved in the responses during the evaluation of kits. The phage clones were isolated by incubating the 12-mer linear random peptide library with the monoclonal antibody (3B3) and VHH antibody (P2) after three rounds of bioscreening. For the two antibodies, a total of 20 phage clones were picked. The inserted DNA fragments of the selected phage clones comprised 36 nucleotides and were translated to 12 amino acid residues. The peptide sequences of the epitopes and the rNS1 protein sequence of DENV2 were aligned and their homology compared. Sequence analysis revealed several different binding epitopes on rNS1 protein identified by all clones but the C-terminal region of amino acids 224–232 was found to be homologous for all clones. For the VHH antibody (P2), seven independent phage clones were obtained after biopanning of the 12-mer library. Among them, ten clones (designated as V1) had the same peptide sequence WHWSYWPGDNRA and another five clones (designated as V2) had the same peptide sequence FHWSWSMQSAAT. Interestingly, these two peptide sequences (WHWSYWPGDNRA and FHWSWSMQSAAT) were the same in both monoclonal and VHH antibodies. When comparing the 12-mer peptide sequence of seven different clones displayed by selected phage binders with the DENV2-rNS1 protein sequence, the peptide sequences for the seven different clones were found to be similar to the sequence of rNS1 amino acids 224–232 (C-terminal region). V1, V2 and V3 (LHWQWWPRQMAS) sequences aligned with the motif 224–232 of rNS1 that had the consensus motif HWxxxxxxW with strongly conserved histidine-tryptophan-tryptophan (Figure 7A).

For monoclonal antibody (3B3), five independent phage clones were obtained after biopanning of the 12-mer library. Fifteen clones designated as M1 had the same peptide sequence WHWSYWPGDNRA and another two clones (designated as M2) had the same FHWSWSMQSAAT peptide sequence. When aligned, the 12-mer peptide sequence of the five different clones displayed by the selected phage binders with the DENV2-rNS1 protein sequence showed identity with the C-terminal region of rNS1 between the amino acids 224–232. Clones M1, M2 and M3 contain the same consensus motif HWxxxxxxW with strongly conserved histidine-tryptophan-tryptophan residues (Figure 7B), which are identical to V1, V2 and V3. One of the amino acid residues is substituted by proline in M5 and V5, which display the consensus motif WPxxxxxW whereas all of the other clones (M4, V4, V6 and V7) have a strongly conserved histidine-tryptophan pair. These results indicate that the C terminal region of rNS1 comprised of amino acids ^224^HWPKPHTLW^232^ is highly conserved for both kinds of antibodies with similar antigenic binding epitopes.

## Discussion

Antibodies are the most preferred tools and are widely used for disease diagnostics and therapy. There is an increasing need for more stable and specific antibodies in the medical and diagnostic fields [37]–[38]. However, conventional hybridoma monoclonal antibody technology is laborious, expensive, time-consuming and has difficulty keeping up with the current pace of recognition of targeted antigen structures. The use of antibody phage display on a llama non-immune library provides a reliable, cost-effective and fast option for generating VHH antibodies. Non-immune libraries have many advantages in that the time-consuming immunization and library construction protocols can be omitted for each new target [39]. VHH antibodies are being researched for multiple pharmaceutical applications and have potential for use in the treatment of some diseases [40]–[42]. This is the first study that has successfully developed a reliable and controllable protocol for nanobody screening against DENV2-rNS1 protein from a non-immune llama VHH library followed by the expression of the nanobodies by *E. coli* (BL21).

After purification, sequence alignment of the VHH antibodies by the IMGT system revealed some sequence homology of the framework regions and residues within the two complementarity determining regions (CDR1, CDR2) with *Lama glama* (IGHV1S3*01, IGHJ4*01). However, the CDR3 regions, previously known to represent a highly antigenic recognition core, were different and did not show homology with the other sequences. Based on these findings, the high affinity of VHH antibodies might be due to these novel and longer CDR3 regions while other CDRs and some contact residues of the framework region may bind opportunistically with the antigen [31]–[32].

The fast, user-friendly and cost-effective immunochromatographic assay, which is based on rapid diagnostic kits for NS1 detection in patient’s serum, provides opportunities for point-of-care diagnosis, especially in developing countries [13], [43]. The selection of well characterized antibodies is crucial for the development of biosensors based on immunoassay. Currently, the frequency of dengue infection has increased because of warmer weather conditions and frequent international travel. On the other hand, few NS1 antigen-based commercial diagnostic kits are available [9], [44]–[48]. Most of these kits are dependent on conventional monoclonal antibodies. In the current study, the discovery of single domain, small-sized (15–16 kDa) and more versatile VHH antibodies with long CDR3 regions offers the potential for the development of a more advanced biosensor.

Moreover, SPR and indirect ELISA results revealed that both VHH and monoclonal antibodies displayed good binding affinity and specificity. P2 VHH antibody was selected due to its higher K_D_ value (2.79×10^–8 ^M) and successful pairing as a capture antibody with 3 M4 detection antibody for the development of a rapid diagnostic kit. The detection limit of the VHH antibody immobilized kit using recombinant NS1 antigen was lower (4.5 ng/ml) than that of the monoclonal antibody immobilized kit (9 ng/ml) (Figure 6). Previously, it has been reported that the concentration of circulating NS1 in acute phase serum samples ranges from 10 ng/ml to 50 µg/ml [8]. The present analytical sensitivity indicates that both kinds of kits are suitable for the detection of low concentrations of NS1 in DENV2 infections. Our findings are supported by some recent studies in which the analytical sensitivities of dengue early rapid tests [14] and NS1 antigen capture ELISA [13] based on monoclonal antibodies were found to be less than 5 ng/ml and 3 ng/ml, respectively, when purified recombinant NS1 was used.

Due to the unavailability of DENV2 positive serum samples from hospitals, the performances of both VHH and monoclonal antibodies immobilized kits were compared with the SD Bioline Dengue Duo commercial kit (Korea) for accuracy of diagnosis. The sensitivity of the monoclonal antibody immobilized kit was consistent with that of the commercial kit with a 1∶150 dilution limit for the detection of native NS1 in DENV2 culture supernatant whereas the VHH immobilized kit displayed a higher sensitivity with a 1∶200 dilution limit. The specificities of the two kits prepared in the present study were also compared with the commercial kit. The specificity of VHH antibody immobilized kit was higher (99.50%) than that of the monoclonal antibody immobilized kit (95.24%) and the commercial kit (98.04%). This comparison study of VHH and monoclonal antibody immobilized rapid diagnostic kits with the commercial kit revealed that kits developed in the present study are reliable and fast. Because of the high target specificity, affinity and ease of production of VHH antibody, the rapid diagnostic kit based on VHH antibody was found to be more efficient and economical and it displayed better sensitivity and specificity results compared to the monoclonal antibody immobilized kit.

Epitope mapping is crucial for understanding the nature of immune responses and diagnostics [49]. Identification of the epitopes requires the preparation of highly specific antibodies. After the successful generation of MAb and VHH antibodies against DENV2-rNS1, the antigenic epitopes were identified by biopanning of a commercially available phage display peptide library (Ph.D 12 kit) using P2 and 3B3 antibodies as targets. After three rounds of panning, phage clones capable of binding to both antibodies were enriched, showing an increase in titer in the case of the VHH antibody compared to the monoclonal antibody. Sequence analysis revealed that there was only one rNS1 epitope residue (corresponding to residues 224–232), which was identical for all clones in both kinds of antibodies. The two peptide sequences WHWSYWPGDNRA (same in V1 and M1 clones) and FHWSWSMQSAAT (same in V2 and M2 clones) were predicted to be the mimotopes on rNS1. The homologous region of the rNS1 sequence (aa224–232) was assessed as a potential epitope for both kinds of antibodies, which indicates that histidine and tryptophan are probably key components in almost all of the clones and form the antigenic epitope (Figure 7A, 7B). These amino acids may play an important role in antibody binding. Similarly, Wu et al. [50]–[51] used serotype-specific monoclonal antibodies to identify the epitopes of DENV1-NS1 and DENV2-NS1 from a random peptide library displayed on a phage. They also found that histidine was the most important amino acid for antibody binding. In consequence, this study revealed that the rNS1 epitope residues ^224^HWPKPHTLW^232^ in the C-terminus epitope region are conserved for both monoclonal and VHH antibodies with similar antigenic epitopes.

In conclusion, selection of VHH against DENV2-rNS1 from a non-immune llama library by a phage display technique proved to be very successful within 4 weeks. Furthermore, VHH expression from *E. coli* and purification within 1 week holds great promise for future large scale production in a cost-effective way. Compared to hybridoma technology, this protocol requires less time and no animal immunization. Selection and screening of positive VHH clones are also accurate and fast. Thus, extensive isolation and production of single domain antibodies become affordable. The comparison of monoclonal and VHH antibodies for the identification and screening of NS1 epitopes may provide better information for developing serological tests. Both kinds of antibodies have similar NS1 epitopes that might be involved in the diagnosis of DENV2 infections. There is no big difference between the sensitivity and specificity of VHH and monoclonal antibody immobilized kits, but better visible results with the VHH antibody immobilized kit indicate that VHHs are more versatile and suitable for binding the pocket or cleft of targeted antigens due to their long CDR3 regions as compared to monoclonal antibodies. All of these findings indicate that VHH offers similar or better results than conventional monoclonal antibody. Therefore, VHH antibodies offer a promising alternative for developing commercial diagnostic tools. One limitation of the present study is the unavailability of DENV2 positive serum samples from hospitals. Further work is still needed to determine the sensitivity of the rapid diagnostic kits with a large number of dengue positive serum samples collected at different times in the course of infection.
